# Media Consumption, Stress and Wellbeing of Video Game and eSports Players in Germany: The eSports Study 2020

**DOI:** 10.3389/fspor.2022.665604

**Published:** 2022-02-14

**Authors:** Kevin Rudolf, Markus Soffner, Peter Bickmann, Ingo Froböse, Chuck Tholl, Konstantin Wechsler, Christopher Grieben

**Affiliations:** Movement-Oriented Prevention and Rehabilitation Sciences, Institute of Movement Therapy and Movement-Oriented Prevention and Rehabilitation, German Sport University Cologne, Cologne, Germany

**Keywords:** gaming, health, media usage, physical activity, sleep

## Abstract

The popularity of video gaming and eSports is increasing rapidly. However, most research focuses on the economical features and psychological consequences of gaming and only little is known about the health behavior of the players. Therefore, this study is a follow-up of the eSports Study 2019 and further investigates the health and health behavior of video game and eSports players in Germany. This cross-sectional study, conducted between April and September 2019, includes 1038 players (91.2% male; 23.0 ± 5.4 years; body mass index: 24.8 ± 5.0 kg/m^2^) who provided data regarding their health status, physical activity, sleep, media consumption, stress and wellbeing via a web-based survey. Descriptive statistics were performed on all questions. Linear regressions were used to examine the relation between media consumption, wellbeing and stress. Almost all respondents classified their health status as “good” or better (92.5%). The average sedentary and physical activity time was 7.2 ± 3.5 h/day and 8.8 ± 10.7 h/week, respectively. Respondents slept for 7.5 ± 1.3 h/night on weekdays and for 8.5 ± 1.5 h/night on weekends, but many were “sometimes” or more frequently overtired (53.1%). Daily duration of playing video games (230.4 ± 159.3 min/day) and watching livestreams and videos with (102.6 ± 101.7 min/day) and without gaming content (72.9 ± 88.5 min/day) were much higher than watching regular television (18.9 ± 49.1 min/day) or reading analog media (32.1 ± 53.5 min/day). In terms of stress and wellbeing, most players reported low stress levels (13.8 ± 5.7) and reached a moderate average score of 60.1 ± 16.4 out of 100 points in the WHO-5 Well-Being Index. Linear regressions revealed no relevant significant associations. The results indicate good subjective health and health behavior of the target group. However, the high amounts of screen-based media-consumption, as well as the moderate stress and wellbeing levels show potential for improvement. In addition, the target group consumed high amounts of digital media in reference to gaming, while traditional media consumption was distinctly low. Consequently, media campaigns that address health promotion in this target group should use the platforms of digital media instead.

## Introduction

Video gaming and competitive video gaming, also known as eSports, have been gaining global attention in recent years. Various structures such as federations and organizations are emerging, global companies are investing in eSports and general gaming, as well as eSports, is becoming increasingly popular in society as a recreational activity. The numbers of the global eSports audience and active eSports players are rising every year (Newzoo, 2020b). According to a current market report (Newzoo, 2020a), the global eSports audience will grow to 495 million people in 2020. The current COVID-19 pandemic could accelerate this trend: most recent studies show a significant increase in gaming and screen-based activities during the pandemic (Colley et al., 2020; DAK Gesundheit, 2020).

The growing interest in video gaming and eSports has also resulted in some significant downsides. Due to the potentially addictive characteristics of excessive gaming, the World Health Organization (WHO) came to include gaming disorder as a diagnosable disorder in the 11th International Classification of Diseases (ICD-11) in 2018 (World Health Organization, 2020). Further issues with video gaming include the large amounts of sitting and screen time associated with the activity (Twenge and Campbell, 2018; DiFrancisco-Donoghue et al., 2019). Sitting and screen time are already recognized as risk factors for numerous chronic diseases (Biswas et al., 2015; Patterson et al., 2018; Bailey et al., 2019) and all-cause mortality (Chau et al., 2013; Biswas et al., 2015; Rezende et al., 2016; Patterson et al., 2018). Sitting (Lissak, 2018) and screen time (Reid Chassiakos et al., 2016; Akçay and Akçay, 2018) also show negative impacts on sleep behavior. While a sedentary lifestyle can lead to sleep disturbances and insomnia (Yang et al., 2017), the blue light emission of screens can influence the circadian rhythm (Hatori et al., 2017), which results in poor sleeping patterns and impoverished health-related quality of life (Mireku et al., 2019). Moreover, prolonged screen time is associated with poor stress regulation (Lissak, 2018), which is additionally a reason for poor sleep (Âkerstedt, 2006). On the positive side, playing video games can also contribute to relaxation (Hoffman and Nadelson, 2009), reduce stress and improve mood (Russoniello et al., 2009).

One question is whether screen-based video gaming and eSports have a negative impact on players' health and sleep *per se*. In order to provide specific health promotion to the target group, collecting data about the current situation of video game and eSports players in terms of health behavior and health status is pivotal. Hence, the aim of this exploratory study is to investigate the media usage, sleep behavior, stress and wellbeing of video game and eSports players and its association with health.

## Materials and Methods

### Study Design and Setting

The current study is a follow-up of the eSports Study 2019 (Rudolf et al., 2020) and was carried out as a cross-sectional online survey. The survey was distributed via the project-related website (esportwissen.de, 2017), social media (*Facebook, Instagram, Discord*), various video game and eSports related platforms, as well as in-person during live events (*ESL One Cologne, ESL Spring Championship, L'Oréal Charcoal Cup*). In addition, the authors' contacts to eSport organizations were used to further disseminate the study. In order to recruit potential participants, an explanation of the aim of the study, eligibility criteria and an incentive (*chance to win one out of five* €*25 Amazon vouchers*) were given during the distribution. The data collection took place from April 5 to September 13, 2019.

The ethical committee of the German Sport University Cologne approved the study (reference: 053/2018).

### Participants

Participants were eligible for the study if they were living in Germany, understood the German language and were actively playing video games or eSports. At the beginning of the survey, an information sheet specifying the purpose of the study was displayed and consent to participate was obtained. Afterwards, a filter question was displayed to verify the current country of residence. For participants who did not live in Germany, the survey ended and they were excluded.

### Measures

The questionnaire was designed to examine video game and eSports players in terms of demographics, video gaming behavior, media usage, health behavior, stress and wellbeing. It was administered via the online survey tool Unipark (*Questback GmbH, Cologne, Germany*). The questionnaire contained a total of 41–43 questions, depending on participants' answers to the filter questions.

Initially, demographic data such as age, gender, education and employment status of the participants, as well as information on body weight and height were collected. Wording and rating of these questions were designed in accordance with the standards of the German Federal Statistical Office (Beckmann et al., 2016).

The questions regarding the data on gaming behavior and media usage were self-designed, considering that no appropriate and validated questionnaire was available. Participants were asked to first classify their player status as:

- Professional players (regularly earning significant revenue from eSports, such as prize money, sponsors, salary from clubs),
- former professionals (used to be an eSport professional, but are no longer),
- amateurs (playing eSports, but without earning a significant amount of money),
- regular players (playing video games or eSports more than once a week, but without taking part in official tournaments and leagues),
- occasional players (playing video games several times a month or less, and without participating in official tournaments and leagues),
- non-players.

The non-players were excluded from all analyses. If participants were amateurs, former professional players or professional players, they received an additional question about their income from price money or player salaries in the last 12 months:

- “no income,”
- “1–500€,”
- “501–1,000€,”
- “1,001–2,500€,”
- “2,501–5,000€,”
- “5,001–10,000€,”
- “more than 10,000€,”
- “prefer not to say.”

Furthermore, all players were asked about their favorite eSports genre (semi-open-ended question) and had to specify how much time they usually spent on the following media each day (hours and minutes):

- “playing eSports games,”
- “playing other video games,”
- “watching live-streams and videos on demand (VOD) with gaming-related content,”
- “watching live streams and videos on demand (VOD) without gaming-related content,”
- “communicating via messenger services or voice chat,”
- “using social media,”
- “browsing the web,”
- “watching television,”
- “listening to music/radio,”
- “reading print media.”

To avoid excessive entries, the hours were limited to 23 and the minutes to 59 in this section.

The questions on health behavior refer to the last 4 weeks. These include:

- Overall health status (5-point rating scale: “poor,” “fair,” “good,” “very good,” “excellent”),
- Duration of moderate to vigorous physical activity (hours per week),
- Duration of sedentary time (hours per day),
- Average time of falling asleep on weekdays and the weekends (hours and minutes),
- Average time of getting up on weekdays and the weekends (hours and minutes),
- Number of awakenings during the night.

Physical activity was described as activities in which the pulse rate increased at least slightly or participants became slightly out of breath. Examples given were cycling, fitness training, playing soccer or climbing stairs. This includes physical activities at work as well as during leisure time. Sedentary time was recorded as sitting or resting, excluding nighttime sleep.

Concluding this topic, participants were asked how often the following statements about behaviors and emotional states with reference to gaming and eSports applied to them (7-point rating scale: “never,” “rarely,” “sometimes,” “several times,” “often,” “very often,” “always”). Wording and rating of these questions were designed with reference to the *EBF-24 A/3* (Kallus and Kellmann, 2016):

- “went to bed later than planned because of the game,”
- “thought about the game during the day,”
- “thought about the game when going to sleep,”
- “got tired of the game,”
- “had a good time with friends in the game,”
- “had a good time with friends away from the game,”
- “slept restlessly,”
- “were overtired,”
- “couldn't fall asleep because of the game.”

The last part of the questionnaire consisted of the entire versions of the *Perceived Stress Scale* (PSS; Cohen et al., 1983) and WHO-Five Well-Being Index (WHO-5; WHO, 1998). While the PSS measures the individual stress perception, the WHO-5 assesses individual wellbeing. Both questionnaires were demonstrated to be valid and reliable tools for research (Topp et al., 2015; Bastianon et al., 2020).

### Data Analyses

Data were examined for completeness, consistency and plausibility. Participants were excluded from all analyses if their data was inconsistent (e.g., reporting to be a high school student with a Bachelor degree) or implausible (reporting daily sleep time <4 or > 16 h). One missing value was allowed for each of the topics on demographics, video gaming behavior, media usage and health behavior, but the respective participants were excluded from the analyses of the respective question. Missing values within the PSS and WHO-5 resulted in exclusion of the participants from the analyses of the respective topic. Participants who did not provide information on height or weight were excluded from all analyses.

The body mass index (BMI) was calculated weight and height of the participants. Additionally, the duration of “playing eSports games” and “playing other video games” was summarized as “active gaming overall”. The sleep duration on weekdays and weekends was calculated for everyone based on the respective time of getting up and going to bed.

Following the manual, the points from the WHO-5 were summed up and multiplied by four to obtain the final score (Mental Health Services, n. d.). Whilst a higher WHO-5 score indicates a healthier wellbeing of the person, a value below 50 marks questionable scores and further medical examinations should be sought (Topp et al., 2015). Scores of the PSS questionnaire were calculated according to the manual (Cohen et al., 1983; Klein et al., 2016).

Descriptive statistics were applied to all data. For each player status, numerical data were described by means and standard deviations, ordinal and nominal data by frequencies. All data were checked for normal distribution. If a non-normal distribution was present, statistical differences between the groups or between two time points were calculated by the Kruskal-Wallis-test, Fisher's exact test or Wilcoxon-test. Otherwise, an ANOVA was performed. *Post-hoc* tests with Bonferroni-correction were performed if overall group differences were statistically significant. Multiple linear regressions were performed to investigate possible associations between the different forms of media usage and wellbeing, stress and duration of sleep on weekdays and weekends. Furthermore, multiple linear regressions were performed between lifestyle factors (e.g., total screentime, sleep data, physical activity) and wellbeing, and subjective health status. For this purpose, media usage variables with screentime were summarized and health status was recoded into a dichotomous characteristic with the parameters “good or better” and “bad or worse”. The level of significance for all analyses was set at *p* < 0.05. All statistical analyses were conducted using IBM SPSS Statistics 26 (IBM Corp., Armonk, NY, USA).

## Results

### Participants

**Figure 1:**
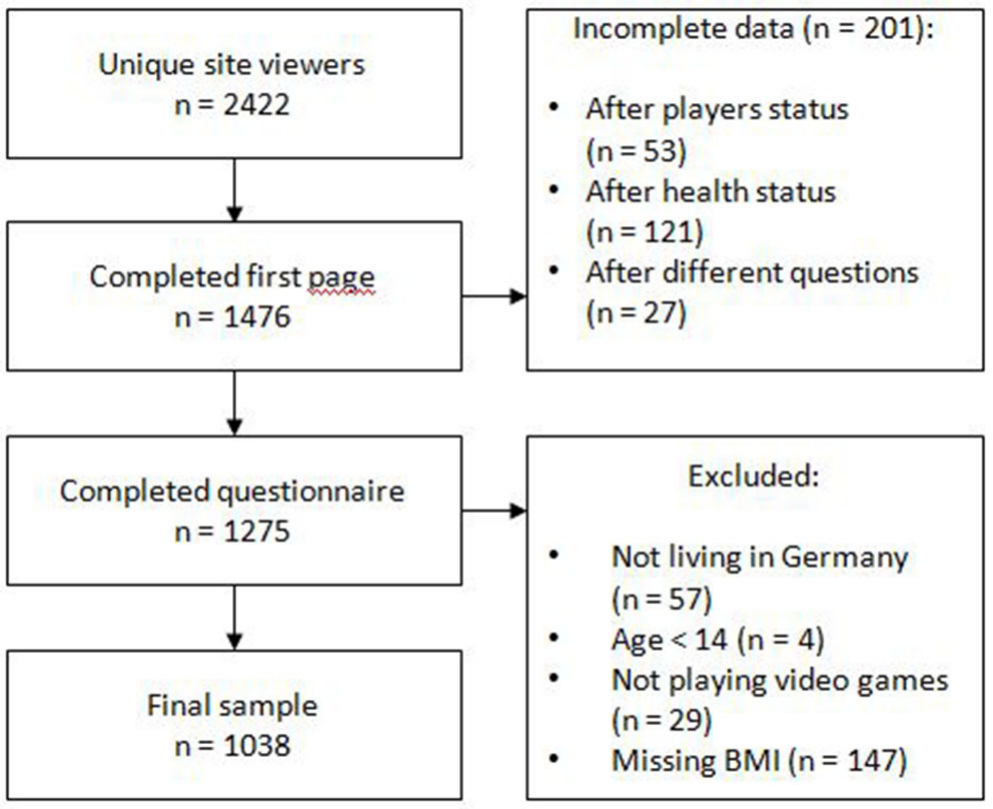
Flow chart of the participation progress.

The flow chart of the participation progress is depicted in Figure 1. Thus, the total sample consists of 1,038 (42.9% of all unique site viewers) participants, of which 26 are professional players, 36 former professional players, 282 amateurs, 545 regular players and 149 occasional players (see Table 1).

**Table 1:** Sample characteristics.

**Group**	**Age^a^ (years) Mean (SD)**	**Gender^b^ (“male”) *n* (%)**	**BMI^a^ (kg/m^2^) Mean (SD)**	**Education^b^ (“higher education entrance qualification or higher”) *n* (%)**	**Employment^b^ (“full-time employment”) *n* (%)**
Total sample (*n* = 1,038)	23.0 (5.4)	947 (91.2)	24.8 (5.0)	569 (54.9)	343 (33.0)
Professional players (*n* = 26)	20.9 (3.3)^O^, ^F^	24 (92.3)	23.9 (4.2)	14 (52.9)	3 (11.5)^F^, ^R^, ^O^
Former professional players (*n* = 36)	26.6 (4.2)^P^, ^A^, ^R^	36 (100.0)^O^	26.4 (5.3)	19 (52.8)	21 (58.3)^P^, ^A^, ^R^, ^O^
Amateurs (*n* = 282)	22.1 (4.7)^O^, ^F^	274 (97.2)^R^, ^O^	25.1 (5.3)	152 (53.9)	79 (28.0)^F^, ^O^
Regular players (*n* = 545)	22.9 (5.5)^O^, ^F^	495 (90.8)^A^, ^O^	24.7 (5.2)	292 (53.6)	183 (33.6)^P^, ^F^
Occasional players (*n* = 149)	24.5 (5.9)^P^, ^A^, ^R^	118 (79.2)^F^, ^A^, ^R^	24.5 (4.0)	92 (61.7)	57 (38.3)^P^, ^F^, ^A^
Sig.	<0.01	<0.01	0.10	0.49	<0.01

a *Kruskal-Wallis test*.

b *Fisher's exact test. Superscript letters indicate statistically significant (p < 0.05) differences to other groups in the same column:*

P *professional players;*

F *former professional players;*

A *amateurs;*

R *regular players;*

O *occasional players*.

### Demographic Data

Table 1 displays the demographic data of the remaining participants, classified by player status. The majority of participants in the sample were between 20 and 30 years old, predominantly male (*n* = 947; 91.2%) and well-educated. Two thirds of the participants were university students (*n* = 300; 28.9%) or working in full-time jobs (*n* = 343; 33.0%), while the other third were pupils (*n* = 151; 14.5%) and apprentices (*n* = 143; 13.8%). Overall, the average BMI of the sample was 24.8 m^2^/kg, which can be classified as normal weight.

### Health Behavior

Most of the participants reported an “excellent” (*n* = 130; 12.8%), “very good” (*n* = 397; 39.0%) or “good” (*n* = 414; 40.7%) health status. Only a few had a “fair” (*n* = 74; 7.3%) or “poor” (*n* = 3; 0.3%) health status (Table 2).

**Table 2:** Data on players' health behavior.

**Group**	**Health status^a^ (Mode) *n* (%)**	**Physical activity^b^ (hours/week) Mean (SD)**	**Sedentary time^a^ (hours/day) Mean (SD)**
Total sample (*n* = 1,018^*^)	“good” 414 (40.7)	8.8 (10.7)	7.2 (3.5)
Professional players (*n* = 26)	“good” 12 (46.2)	7.3 (7.7)	6.2 (4.3)
Former professional players (*n* = 36)	“good” 15 (41.7)	10.3 (13.2)	7.0 (3.5)
Amateurs (*n* = 276)	“good” 115 (40.8)	8.7 (9.6)	7.3 (3.6)
Regular players (*n* = 534)	“good” 220 (40.4)	9.0 (11.4)	7.3 (3.4)
Occasional players (*n* = 146)	“very good” 69 (46.3)	8.5 (9.9)	7.0 (3.5)
Sig.	0.90	0.83	0.46

a *Kruskal-Wallis Test*.

*^2^ANOVA*.

* *In these three categories, 20 data sets were incomplete*.

Overall, the participants reported to be physically active for about 8.8 (± 10.7) hours per week and the majority (*n* = 819; 80.5%) exceeded the WHO recommendations of 2.5 h of physical activity per week. The average amount of sedentary time was 7.2 (± 12.4) hours per day. No statistically significant differences between player groups were found in the data on health behavior.

Table 3 presents the results on sleeping patterns. While the total sample spent an average of 7.5 ± 1.3 h/night asleep on weekdays, occasional players (7.9 ± 1.1 h/night) slept significantly longer than former professionals (7.1 ± 1.5 h/night; *p* = 0.04), amateurs (7.4 ± 1.4 h/night; *p* < 0.01) and regular players (7.5 ± 1.3 h/night; *p* < 0.01). On weekends (8.5 ± 1.5), the total sample slept ~1 h longer than on weekdays (7.5 ± 1.3, *p* < 0.01). During the night, all participants woke up more than once on average, and the regular players (1.2 ± 1.9 h/night) woke up significantly less often than the occasional players (1.6 ± 2.1 h/night; *p* = 0.02).

**Table 3:** Sleep outcomes.

**Group**	**Sleep time weekdays^a^ (hours/night) Mean (SD)**	**Sleep time weekends^a^ (hours/night) Mean (SD)**	**Waking up at night^a^ Mean (SD)**	**Restless sleep^a^ (Mode) *n* (%)**	**Overtired^a^ (Mode) *n* (%)**
Total sample (*n* = 937^*^)	7.5 (1.3)	8.5 (1.5)	1.3 (2.2)	“rarely” 384 (41.0)	“rarely” 314 (33.5)
Professional players (*n* = 21)	7.9 (1.7)	8.4(1.4)	1.3 (2.4)	“rarely” 10 (47.6)	“rarely” 7 (33.3) “sometimes” 7 (33.3)
Former professional players (*n* = 32)	7.1 (1.5)^O^	8.0 (1.4)	1.3 (1.6)	“rarely” 15 (46.9)	“rarely” 11 (34.4)
Amateurs (*n* = 246)	7.4 (1.4)^O^	8.4 (1.5)	1.4 (2.8)	“rarely” 93 (37.8)	“rarely” 87 (35.4)
Regular players (*n* = 502)	7.5 (1.3)^O^	8.5 (1.5)	1.2 (1.9)^O^	“rarely” 207 (41.2)	“rarely” 175 (34.9)
Occasional players (*n* = 136)	7.9 (1.1)^a^, ^R^, ^F^	8.6 (1.3)	1.6 (2.1)^R^	“rarely” 59 (43.4)	“sometimes” 46 (33.8)
Sig.	<0.01	0.09	0.02	0.54	0.54

a *Kruskal-Wallis; Superscript letters indicates statistically significant (p <0.05) differences to other groups in the same column*.

*^P^professional players;*

F *former professional players;*

*^A^amateurs;*

R *regular players;*

O *occasional players*.

* *In these five categories, 70 data sets were inconsistent or incomplete*.

With regards to the information on restless sleep, the majority of players specified “never” (*n* = 271; 28.9%), “rarely” (*n* = 384; 41.0%) or “sometimes” (*n* = 170; 18.1%). Furthermore, about half reported that they were “sometimes” (*n* = 264; 28.2%) or more often (*n* = 234; 24.9%) overtired.

### Media Usage

**Figure 2:**
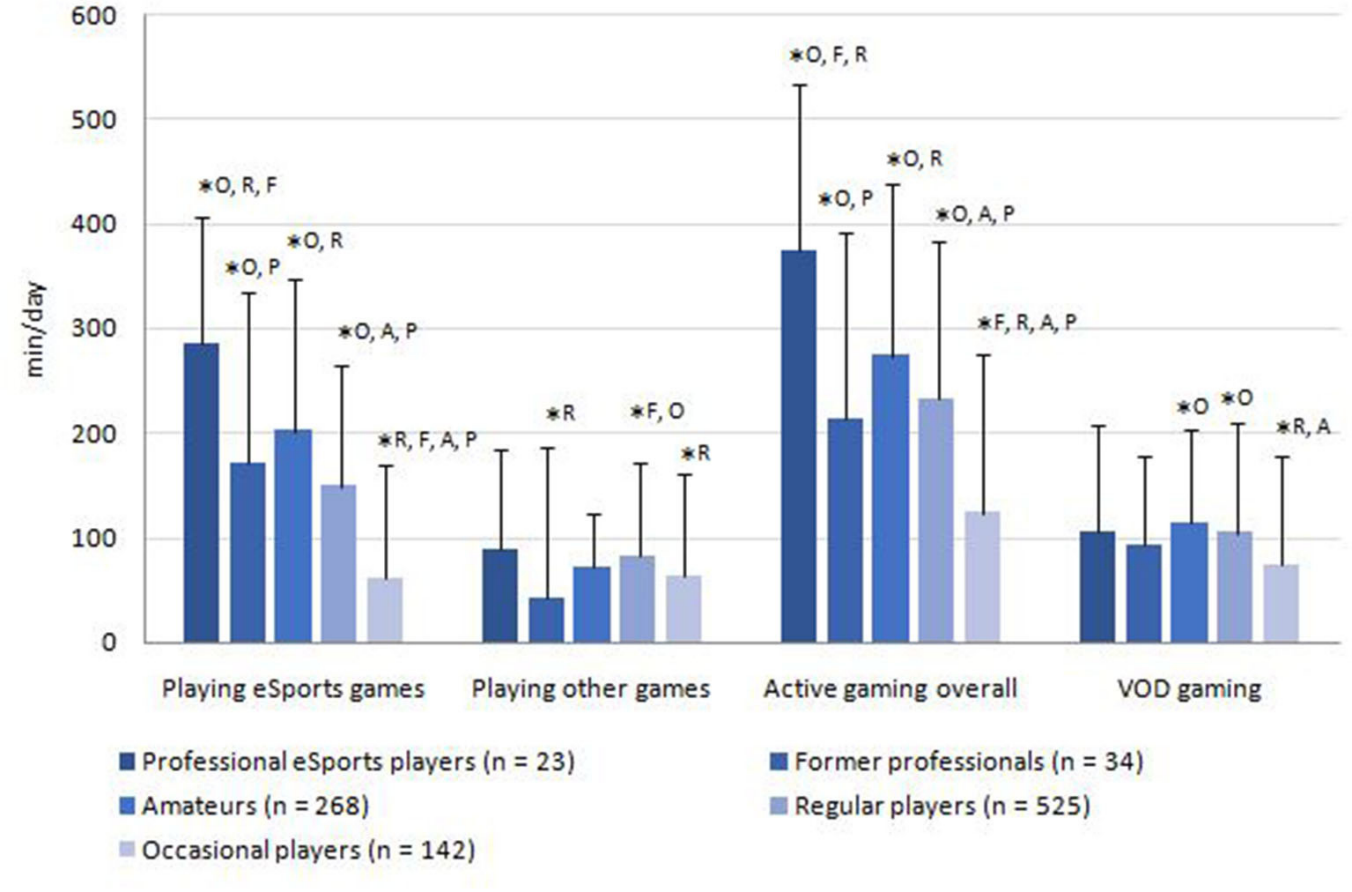
Average consumption of gaming related media per day. *Superscript letters indicate statistically significant (*p* < 0.05) differences to other groups by Kruskal-Wallis test. ^P^professional players; ^F^former professional players; ^a^amateurs; ^R^regular players; ^O^occasional players. VOD, Live-streams/videos on demand.

**Figure 3:**
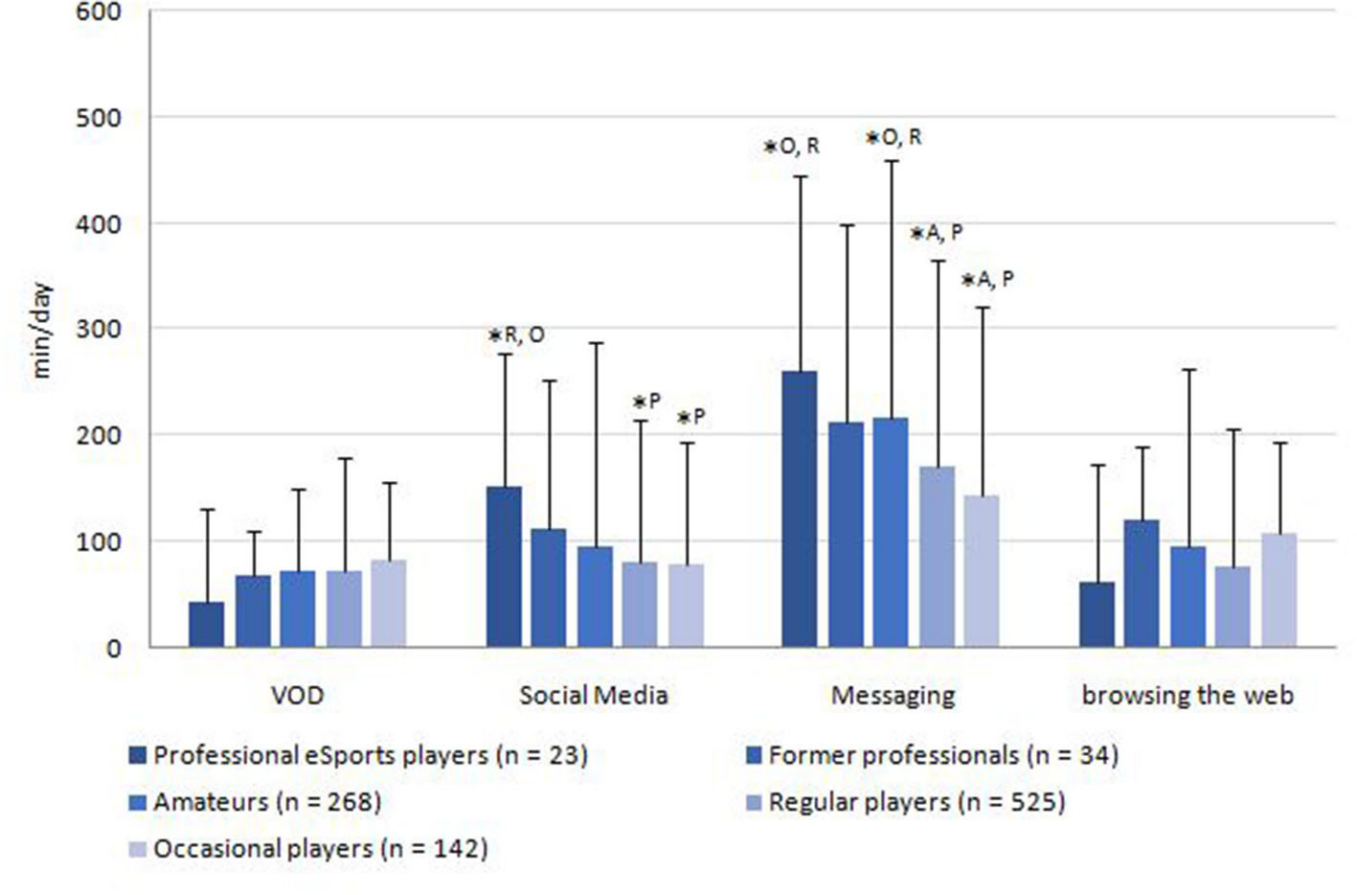
Average consumption of online media without gaming reference per day. *Superscript letters indicate statistically significant (*p* < 0.05) differences to other groups by Kruskal-Wallis test. ^P^professional players; ^F^former professional players; ^a^amateurs; ^R^regular players; ^O^occasional players. VOD, Live-streams/videos on demand.

**Figure 4:**
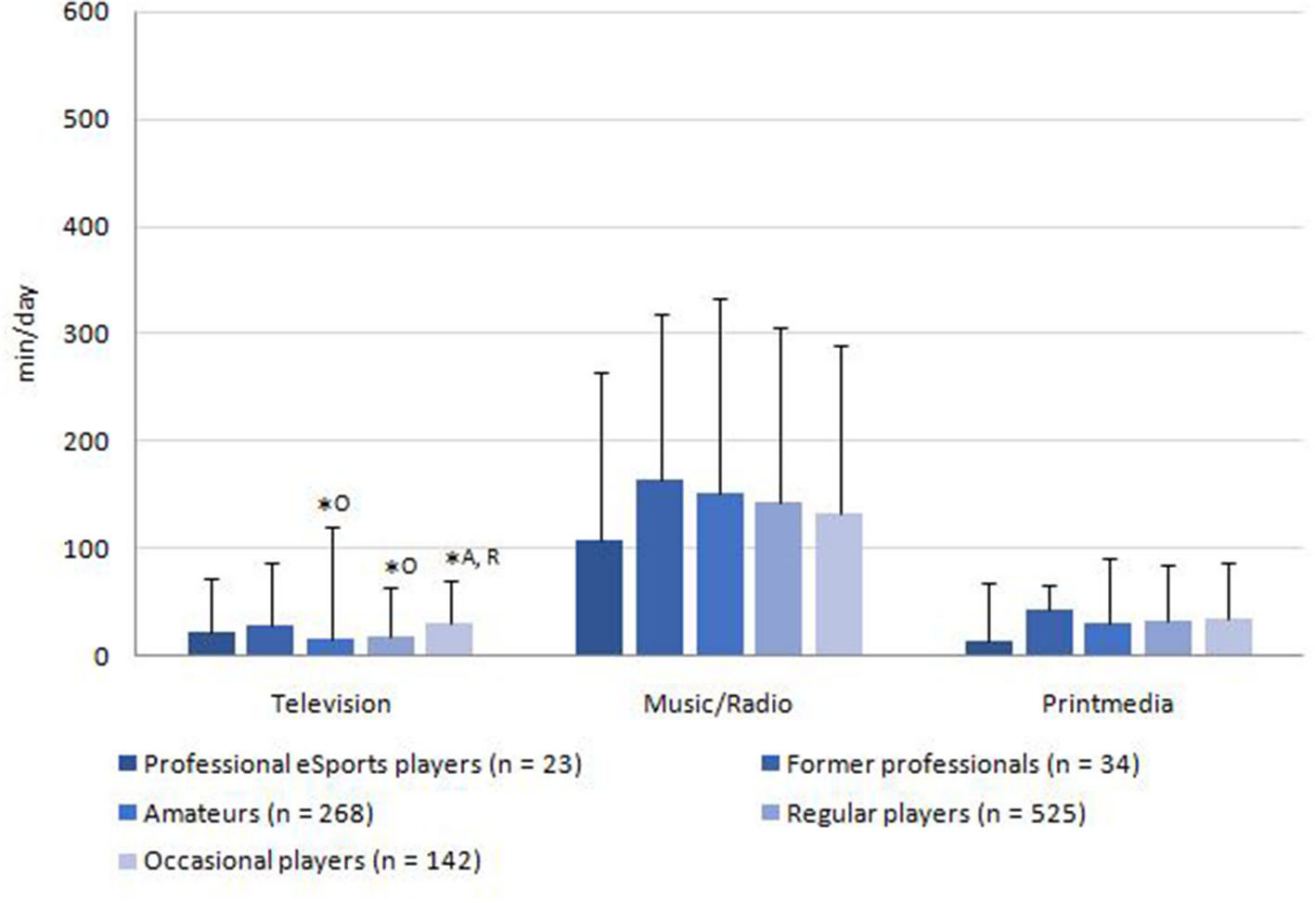
Average consumption of traditional media without gaming reference per day. *Superscript letters indicate statistically significant (*p* < 0.05) differences to other groups by Kruskal-Wallis test. ^P^professional players; ^F^former professional players; ^a^amateurs; ^R^regular players; ^O^occasional players.

The media usage of the sample is displayed in Figures 2–4. The average total media usage time of professional eSports players was the highest (1,140.0 ± 474.8 min/day). However, only the usage time of occasional players (806.2 ± 564.0 min/day) was statistically significantly lower than those of all other player groups (*p* < 0.05). Additionally, the amateurs (1,066 ± 610.2 min/day) spent significantly more time on media than the regular players (928.6 ± 526.0 min/day; *p* = 0.019).

The most frequented medium was active gaming overall (230.4 ± 159.3 min/day). The favorite game genres were *tactical shooters*, followed by *multiplayer online battle arenas* and *sports and racing simulations* (Table 4).

**Table 4:** Prioritized game genres (*n* = 1,038).

**Game genres**	**Frequency *n* (%)**
Tactical shooters	581 (56.0)
Multiplayer online battle arenas	197 (19.0)
Sport and racing simulations	90 (8.7)
Battle royales	58 (5.6)
Massively multiplayer online role playing games	28 (2.7)
Real time strategy games	21 (2.0)
Collecting card games	12 (1.2)
Mobile games	12 (1.2)
Fighting games	4 (0.4)
Others	35 (3.4)

Regarding the time spent with gaming, statistically significant differences were found between the player groups (see Figure 2). For example, eSports professionals played the most, especially eSports games (285.8 ± 16.7 min/day). In relation to former professionals (171.6 ± 146.0 min/day; *p* = 0.04) and the regular players (60 ± 85.3 min/day; *p* < 0.01), they played significantly more eSports games. Concerning watching VODs with gaming reference, only the occasional players (74.3 ± 84.8 min/day) differed from the regular players (104.6 ± 104 min/day; *p* < 0.001) and amateurs (114.6 ± 105.7 min/day; *p* < 0.001).

As seen in Figure 3, the time spent with VODs without gaming reference (72.9 ± 88.5 min/day) is lower than with gaming reference (102.5 ± 101.7 min/day; *p* < 0.001). Regarding the online non-gaming media, messaging accounts for the highest time span (182.5± 185.2 min/day), followed by the use of social media (86.6 ± 124.6 min/day). The professional players (260 ± 186.6 min/day) communicated via messenger services significantly more often than the occasional players (170.2 ± 179.5 min/day; *p* = 0.05) and regular players did (142.5 ± 155.1 min/day; *p* = 0.01). Likewise, the use of social media was highest among professional players (152.0 ± 141.8 min/day) and differed significantly from occasional (80.6 ± 116.5 min/day; *p* = 0.01) and regular players' usage (78.1 ± 109.8 min/day; *p* = 0.01).

Traditional media consumption was the lowest in the sample (Figure 4). While music and radio (143.8 ± 156.3 min/day) were consumed approximately as much as online media, watching television (18.9 ± 49.1 min/day) and reading analog media (32.1 ± 53.5 min/day) scored much lower. Statistically significant differences were present between occasional players (60.63 ± 85.3 min/day) and regular players (149.2 ± 109.3 min/day; *p* = 0.02) and amateurs (15.2 ± 46.5 min/day; *p* = 0.01).

### PSS and WHO-5

Table 5 displays the results of the WHO-5 and PSS questionnaires. No statistically significant differences between the player groups were found in both questionnaires.

**Table 5:** Scores and classifications of the WHO-5 and PSS questionnaire.

	**WHO-5 Score^a^ (1–100) Mean (SD)**	**PSS Score^a^ (0–40) Mean (SD)**
Total sample (*n* = 1,038)	60.1 (16.4)	13.8 (57)
Professional players (*n* = 26)	62.2 (16.1)	14.0 (5.4)
Former professional players (*n* = 36)	61.6 (19.3)	13.3 (5.3)
Amateurs (*n* = 282)	61.1 (15.3)	13.0 (5.5)
Regular players (*n* = 545)	60.1 (16.5)	14.0 (5.6)
Occasional players (*n* = 149)	57.9 (16.9)	14.6 (6.3)
Sig.	0.08	0.14

a *Kruskal-Wallis Test*.

### Associations With Media Usage

Statistically significant minor associations between media usage and the sleep time on weekdays [F_(13, 935)_ = 3.89, *p* < 0.001] as well as on weekends [F_(13, 948)_ = 2.81, *p* < 0.01] were found (Tables 6–9). Accordingly, sleep was 6 min shorter on weekdays for every hour of non-eSports video game time. Additionally, the participants slept 6 min less for every hour they were using social media on weekdays. In contrast, sleep was 6 min longer for every hour of watching VODs without gaming reference. During weekends, the participants slept 6 min less for every hour of browsing the internet.

**Table 6:** Multiple regression: media usage in relation to sleep on weekdays.

**Variables**	**B**	**SE**	**Beta**	**95% CI (lower; upper limit)**	**Sig**.
Playing eSport games (min/day)	−0.04	0.03	−0.05	−0.10; 0.01	0.14
Playing other video games (min/day)	−0.109	0.03	−0.09	−0.16; −0.03	0.01
Watching live-streams and videos (VOD) with gaming related content (min/day)	0.03	0.03	0.03	−0.04; 0.09	0.44
Watching live streams and videos (VOD) without gaming-related content (min/day)	0.09	0.04	0.08	0.02; 0.16	0.02
Communicating via messenger services or voice chat (min/day)	−0.01	0.02	−0.02	−0.05; 0.03	0.53
Using social media (min/day)	−0.06	0.03	−0.08	−0.12; < −0.01	0.04
Browsing the web (min/day)	<0.01	0.03	0.00	−0.06; 0.06	0.94
Watching television (min/day)	0.11	0.07	0.06	−0.02; 0.25	0.09
Listening to music/radio (min/day)	−0.03	0.02	−0.05	−0.07; 0.01	0.12
Reading print media (min/day)	<0.01	0.06	<0.01	−0.12; 0.12	0.98
Age (years)	−0.47	0.63	−0.03	−1,71; 0.78	0.46
Gender (male = 1; female = 2)	−5.65	10.83	−0.02	−26.90; 15.60	0.60
BMI (kg/m^2^)	−2.14	0.64	−0.11	−3.39; −0.89	<0.01

*Dependent variable: sleep on weekdays (min/day), n = 949; p <0.001, adjusted R^2^ = 0.038*.

**Table 7:** Multiple regression: media usage in relation to sleep on weekends.

**Variables**	**B**	**SE**	**Beta**	**95% CI (lower; upper limit)**	**Sig**.
Playing eSport games (min/day)	−0.03	0.03	−0.03	−0.09; 0.03	0.34
Playing other video games (min/day)	<0.01	0.04	<0.01	−0.07; 0.08	0.93
Watching live-streams and videos (VOD) with gaming related content (min/day)	<0.01	0.04	<0.01	−0.07; 0.08	0.97
Watching live streams and videos (VOD) without gaming-related content (min/day)	0.02	0.04	0.02	−0.06; 0.10	0.59
Communicating via messenger services or voice chat (min/day)	−0.02	0.02	−0.03	−0.06; 0.02	0.38
Using social media (min/day)	−0.05	0.03	−0.06	−0.11; 0.02	0.13
Browsing the web (min/day)	−0.09	0.04	−0.09	−0.15; −0.02	0.01
Watching television (min/day)	0.09	0.07	0.04	−0.06; 0.23	0.24
Listening to music/radio (min/day)	−0.01	0.02	−0.02	−0.06; 0.04	0.66
Reading print media (min/day)	−0.13	0.07	−0.07	−0.26; <0.01	0.06
Age (years)	−1.74	0.70	−0.09	−3.12; −0.36	0.01
Gender (male = 1; female = 2)	−5.80	12.25	−0.02	−29.85; 18,24	0.64
BMI (kg/m^2^)	−0.04	0.72	< -0.01	−1.46; 1.37	0.95

*Dependent variable: sleep on weekends (min/day), n = 962; p <0.01, adjusted R^2^ = 0.024*.

**Table 8:** Multiple regression: media usage in relation to perceived stress scale score.

**Variables**	**B**	**SE**	**Beta**	**95% CI (lower; upper limit)**	**Sig**.
Playing eSport games (min/day)	<0.01	<0.01	< -0.01	< -0.01; <0.01	0.90
Playing other video games (min/day)	0.01	<0.01	0.13	<0.01; 0.01	<0.01
Watching live-streams and videos (VOD) with gaming related content (min/day)	<0.01	<0.01	0.03	< -0.01; 0.01	0.35
Watching live streams and videos (VOD) without gaming-related content (min/day)	<0.01	<0.01	0.05	< -0.01; 0.01	0.14
Communicating via messenger services or voice chat (min/day)	< -0.01	<0.01	−0.03	< -0.01; <0.01	0.46
Using social media (min/day)	<0.01	<0.01	0.04	< -0.01; 0.01	0.24
Browsing the web (min/day)	<0.01	<0.01	<0.01	< -0.01; <0.01	0.97
Watching television (min/day)	<0.01	<0.01	0.01	< -0.01; 0.01	0.68
Listening to music/radio (min/day)	< -0.01	<0.01	−0.04	< -0.01; <0.01	0.19
Reading print media (min/day)	< -0.01	<0.01	−0.04	−0.01; <0.01	0.27
Age(years)	−0.06	0.04	−0.06	−0.13; 0.01	0.10
Gender (male = 1; female = 2)	3.31	0.65	0.16	2.04; 4.58	<0.01
BMI (kg/m^2^)	0.05	0.04	0.04	0.03; 0.12	0.20

*Dependent variable: perceived stress scale score, n = 992; p <0.001, adjusted R^2^ = 0.044*.

**Table 9:** Multiple regression: media usage in relation to the WHO-5 scores.

**Variables**	**B**	**SE**	**Beta**	**95% CI (lower; upper limit)**	**Sig**.
Playing eSport games (min/day)	<0.01	0.01	0.09	<0.01; 0.02	0.01
Playing other video games (min/day)	< -0.01	0.01	−0.02	−0.01; 0.01	0.64
Watching live-streams and videos (VOD) with gaming related content (min/day)	< -0.01	0.01	−0.02	−0.01; 0.01	0.67
Watching live streams and videos (VOD) without gaming-related content (min/day)	−0.01	0.01	−0.04	−0.02; <0.01	0.20
Communicating via messenger services or voice chat (min/day)	< -0.01	<0.01	−0.03	−0.01; <0.01	0.50
Using social media (min/day)	<0.01	0.01	0.02	−0.01; 0.01	0.52
Browsing the web (min/day)	−0.01	0.01	−0.05	−0.02; <0.01	0.22
Watching television (min/day)	−0.01	0.01	−0.02	−0.03; 0.02	0.53
Listening to music/radio (min/day)	<0.01	<0.01	0.03	< -0.01; 0.01	0.39
Reading print media (min/day)	0.03	0.01	0.08	0.01; 0.05	0.02
Age (years)	−0.02	0.11	−0.01	−0.22; 0.19	0.89
Gender (male = 1; female = 2)	−5.12	1.87	−0.09	−8.79; −1.45	0.01
BMI (kg/m^2^)	−0.27	0.11	−0.08	−0.49; −0.06	0.01

*Dependent variable: WHO-5 score, n = 992; p <0.01, adjusted R^2^ = 0.017*.

Furthermore, statistically significant minor correlations between media usage and the PSS scores were found [F_(13, 978)_ = 4.48, *p* < 0.001]. Therefore, the score of the PSS increased by 4.8 for every hour which the participants spent playing non-eSports video games.

There are also significant correlations between media usage and the WHO-5 [F_(13, 978)_ = 2.36, *p* < 0.01]. In this case, the final score of the WHO-5 increased by 0.6 for every hour the participants spent playing eSport video games, and by 1.8 for every hour spent on analog media.

Statistically significant minor associations between lifestyle factors and wellbeing [F_(9, 939)_ = 5.87, *p* < 0.001] as well as the subjective health status [$\chi_{\left(9\right)}^{2}$ = 68.01, *p* < 0.01] were found (Tables 10, 11). Accordingly, increased sitting time, BMI and sleep on weekends have a small negative impact on wellbeing. Longer sleep on weekdays has a positive impact on the participants' wellbeing. In line with this, longer sitting time and higher BMI also have a small negative impact on subjective health status. In addition, significant associations between a good health status and higher levels of physical activity were found.

**Table 10:** Multiple regression: lifestyle factors in relation to the WHO-5 scores.

**Variables**	**B**	**SE**	**Beta**	**95% CI (lower; upper limit)**	**Sig**.
Total screentime (min/day)	0.00	<0.01	−0.01	< -0.01; <0.01	0.79
Sleep on weekdays (hours)	0.86	0.37	0.08	0.13; 1.59	0.02
Sleep on weekends (hours)	−1.05	0.33	−0.11	−1.70; −0.41	<0.01
Sitting time (hours/day)	−0.43	0.15	−0.09	<0.01; −0.72	<0.01
Physical activity (hours/week)	0.09	0.05	0.06	−0.02; 0.18	0.10
Times awake per night	−0.69	0.23	−0.10	−1.15; −0.23	<0.01
Age (years)	−0.12	0.10	−0.04	−0.32; 0.08	0.23
Gender (male = 1; female = 2)	−5.10	1,78	−0.09	−8.59; −1.61	<0.01
BMI (kg/m^2^)	−0.25	0.11	−0.08	−0.47; −0.04	0.02

*Dependent variable: WHO-5 score, n = 915; p <0.001, adjusted R^2^ = 0.042*.

**Table 11:** Multiple regression: lifestyle factors in relation to the subjective health status.

**Variables**	**B**	**SE**	**Wald**	**95% CI (lower; upper limit)**	**Sig**.
Total screentime (min/day)	0.00	0.00	1.93	1.00; 1.00	0.17
Sleep on weekdays (hours)	0.15	0.09	2.59	0.97; 1.39	0.11
Sleep on weekends (hours)	−0.13	0.08	2.37	0.75; 1.04	0.12
Sitting time (hours/day)	−0.09	0.04	5.83	0.86; 0.98	0.02
Physical activity (hours/week)	0.09	0.03	10.37	1.04; 1.16	<0.01
Times awake per night	−0.10	0.04	4.83	0.84; 0.99	0.03
Age (years)	0.5	0.03	2.70	0.99; 1.11	0.10
Gender (male = 1; female = 2)	−0.61	0.38	2.51	0.26; 1.15	0.11
BMI (kg/m^2^)	−0.11	0.02	25.08	0.86; 0.94	0.03

*Dependent variable: dichotomized subjective health status (“good or better” = 1 vs. “bad or worse” = 0), n = 949; p <0.01, Nagelkerkes R^2^ = 0.165*.

## Discussion

### Key Results

The current study aimed at providing insights into the health behavior and media usage of video game and eSports players in Germany. Data from more than 1,000 players indicate that the general state of health is decent and the vast majority of players claim to be sufficiently physically active, but especially consumption of digital media is high. In terms of sleep, stress and wellbeing, the study shows strikingly positive results, although there may be room for further improvement.

### Demographics

In line with previous studies on gaming and eSports, the results of the present study show that video game and eSport players are predominantly young, male and well-educated (Rudolf et al., 2020). Similar to the average population, the participants had a normal BMI (Statistisches Bundesamt, 2017) and moderate sitting times (Clemes et al., 2016; Froböse et al., 2018). The majority of the sample consisted of students, apprentices and pupils, and, in the context of their current lifestyles, most performed prolonged sedentary activities in their everyday life. Besides, the participants spent many hours playing video games, which is mostly done while sitting. Considering this, the sitting times are actually surprisingly low. Equally positive is the fact that the participants exceeded the WHO recommendations for physical activity of 2.5 h per week (World Health Organization, 2010) by a lot. In comparison to the general German population, more than twice as many percent of participants were sufficiently physically active (Finger et al., 2017). Accordingly, the data disproves the established clichés about video game and eSport players which claim that they are sedentary and physically inactivemost of the time. To substantiate these results, more objective measures of health and activity behaviors like physical examinations, fitness trackers or comparable devices could be included in future research.

### Sleep Outcomes

Video game and eSport players can still improve with regard to sleep and the regeneration phase. Overall, the participants had similar sleep durations as the German population (Feld and Young, 2017), but still do not fulfil the recommendations of 8–10 and 7–9 h per night for teenagers and (young) adults, respectively (Hirshkowitz et al., 2015). In addition, more than the half reported that they were overtired at least some of the time. A persistent fatigue can lead to numerous negative health consequences and the general performance decreases (Fortier-Brochu et al., 2010; Lock et al., 2018), which impacts all areas of life. Reduced cognitive performance, impaired metabolic processes and mental health problems are only some examples (Lock et al., 2018). The impaired sleep patterns may be caused by the blue light emission of screens, which has an influence on the circadian rhythm (Hatori et al., 2017). In our study, a small connection between certain types of media and sleep could be observed. Accordingly, participants slept less when they spent more time playing non-eSports games or dealt with social media. Reduced sleep time, in turn, tended to be associated with lower wellbeing. However, these correlations were only apparent on weekdays. More so, this does not specify any ultimate conclusion about the sleep quality itself. The extent to which associations between video gaming and sleep can be found has not yet been sufficiently proven (Huard Pelletier et al., 2020). A reduction in screen time should be kept in mind, especially before going to bed, as this can affect sleep and wellbeing (Hale and Guan, 2015). These relationships need further research, particularly with regard to sleep quality and differences between weekday and weekend sleep patterns. Nevertheless, the study emphasized that almost the half of the participants are never or only rarely overtired. They may compensate for the lack of sleep during the week during the weekends, considering that participants sleep 1 h longer on average.

### Media Usage

Recent studies show that increased media usage can lead to physical and psychological complaints (Bernath et al., 2020) and has a negative impact on health-related quality of life (Finne et al., 2013). In light of this, reducing screen time can lead to better general and mental health (Colley et al., 2020). However, although very high digital media usage times were the case for all player categories, no relevant correlations with stress perception and wellbeing could be observed in our study. One possible reason might be the long durations of physical activity that might partially balance the negative effects of screen time. Nevertheless, further research is needed to confirm these results and get better insight into the possible interaction of the health behavior and media usage in this target group.

Due to the recruitment and eligibility criteria of the present study, gaming was logically the medium used the most by the participants. Professional gamers spent most of their daily time with gaming, especially eSports games. The particularly high playing time of the professional eSports players is probably due to the fact that gaming is their main source of income and, in this context, they can devote more time to gaming. Moreover, their career depends on their performance, which is why they have to invest more time in training their skills. However, amateurs also reported that they played for many hours a day; even regular and occasional players still played for an average of more than 2–3 h a day. These high amounts of playing time must be viewed critically. Generally, the excessive use of video games is associated with poorer self-reported general health (Wenzel et al., 2009), high energy intake (Lyons et al., 2012) and further negative effects (Mentzoni et al., 2011). Even though these consequences could not be observed in our study, the possible negative effects should still not be ignored.

Additionally, high social media usage and messaging times were apparent across all player categories. The professional eSports players also had the highest usage times of these media. Negative correlations between social media use and for example depression or anxiety have already been proven (Barry et al., 2017). However, the risk for mental health problems is especially increased by a use of social media for more than 3 h per day (Riehm et al., 2019), which none of the player groups reached on average. The participants probably use this medium primarily for networking and communicating with friends and the gaming community, as the particularly high messaging times show. Many players get to know each other online and live all over Germany, which is why other means of networking such as social media come in handy.

Similar to other studies, traditional media consumption such as reading print media is low compared to online media (Beisch et al., 2019; Medienpädagogischer Forschungsverbund Südwest, 2020). The only exception is television, which remains the most used medium in the German population. However, new media like online livestreams are on the rise and are already present in young age groups. Especially in 2020, the online media recorded an increase due to the COVID-19 pandemic (Lemenager et al., 2020; Medienpädagogischer Forschungsverbund Südwest, 2020). The target group investigated in the current study tends to use more online media. Only a very small proportion consumed television and print media. This is a point that could be relevant to media campaigns that address, for example, health promotion in this target group. These campaigns should possibly make use of online media as well, as traditional media are no longer the main focus of the young target group.

### Wellbeing and Stress

In terms of wellbeing, the average score of the participants was above 50, which is considered the cut-off for “questionable” wellbeing (Topp et al., 2015). Hence, the wellbeing of the video game and eSports players can be considered as good. They show similar scores to amateur and professional athletes in traditional sports (Spengler et al., 2013). These positive values go hand in hand with the eSports players' perception of stress. Most eSports players experience low levels of stress. This is also to be found in the average German population (Klein et al., 2016). It is certain that the professional eSports players have many internal and external stressors (Smith et al., 2019) and that so-called media multitasking has an influence on the perceived stress (Reinecke et al., 2017). It is also possible that the aspects of increased media consumption, interaction with the community and therefore the constant social availability of the eSports player have a considerable influence on their stress perception. It is necessary for future research to investigate further the influencing factors of the perceived stress levels of eSports players.

Although regression coefficients were small, the results indicate significant associations between BMI and sitting time and subjective health status. Since physical activity can counteract this potentially negative impact, it is recommended to implement physical activity programs in the training of eSport athletes. In conclusion, despite some areas showing room for improvement, the eSports players reported a very positive general health status. Accordingly, many outdated clichés about eSports players seem to not be the case in our study.

### Limitations

The present study also has some limitations. Due to the data collection at live events, the respective game genres at display during the events may be overrepresented in this sample. To compensate for this, the questionnaire was distributed on various online gaming websites. Nevertheless, about half of the sample chose tactic shooters as their main genre, while market reports indicate that this genre only makes up for about 20% percent of games played (Entertainment Software Association, 2019).

Secondly, most of the topics were only addressed through a small number of questions in order to provide data on a wide range of different health behaviors. In light of this and due to the cross-sectional design of the study, most of the topics were introduced in a purely descriptive manner and no information on causality can be provided. In terms of media usage, it must also be taken into account that many of the media can be used simultaneously, such as watching live streams and chatting via messenger apps or listening to music while playing games (van der Schuur et al., 2015). The questionnaire did not explicitly ask for simultaneous use of media, but incorporating this in future studies could provide a more detailed insight into the total screen time of participants.

Thirdly, many participants were excluded from the study due to missing answers in the BMI variables. The reason for this omission was the decision to provide results on a well-described sample of almost the same size in all calculations. Adding the excluded records does not lead to significant change in the results, so the decision to exclude individuals with missing BMI values was retained.

Lastly, the present study is based exclusively on self-reported data. For this reason, the results can be affected by social desirability or subjective over- and underestimation of certain behaviors like sedentary times or physical activity (Prince et al., 2008; Dowd et al., 2018). In order to minimize these inconsistencies, paradoxical data were excluded and data were checked for extreme values to prevent distortion through erroneous information. However, effects of social desirability and inaccurate reporting cannot generally be excluded.

## Conclusions

Overall, the video game and eSports players show sufficient general health and have predominantly positive health behaviors. Many aspects with the potential for further improvement, such as sleep patterns and the moderate stress in professional players, are also the case in the German general population. A closer look should be taken at the high duration of media usage, which could be increased as digitization progresses and thus have further negative consequences on the players' health. To keep this young target group healthy, it is important that they receive appropriate support. In this respect, it is necessary to use online media to address this group in a target group-oriented manner.

## Data Availability Statement

The raw data supporting the conclusions of this article will be made available by the authors, without undue reservation.

## Ethics Statement

The studies involving human participants were reviewed and approved by the Ethical Committee of the German Sport University Cologne approved the study (reference: 053/2018). Written informed consent from the participants' legal guardian/next of kin was not required to participate in this study in accordance with the national legislation and the institutional requirements.

## Author Contributions

KR, IF, and CG: conceptualization. KR, MS, PB, CT, and KW: data curation. KR and KW: formal analysis. IF and CG: funding acquisition, project administration, and supervision. KR, MS, PB, IF, CT, KW, and CG: investigation and writing—review and editing. KR: methodology. IF: resources. KR, MS, PB, CT, KW, and CG: validation. KR, MS, and IF: visualization. KR and MS: writing—original draft. All authors contributed to the article and approved the submitted version.

## Funding

The present study is part of the esportwissen.de-project, which was financially supported by the AOK Rhineland/Hamburg. The funders had no influence on the design of the study; in the collection, analyses or interpretation of data; in the writing of the manuscript, or in the decision to publish the results.

## Conflict of Interest

The authors declare that the research was conducted in the absence of any commercial or financial relationships that could be construed as a potential conflict of interest.

## Publisher's Note

All claims expressed in this article are solely those of the authors and do not necessarily represent those of their affiliated organizations, or those of the publisher, the editors and the reviewers. Any product that may be evaluated in this article, or claim that may be made by its manufacturer, is not guaranteed or endorsed by the publisher.
